# Case Report: Pseudomonas can take a toll on a patient

**DOI:** 10.12688/f1000research.53424.2

**Published:** 2021-09-21

**Authors:** David K. Buchbinder, Jasjit Singh, Tuan Dao, Aaron Sassoon, Antonio Arrieta

**Affiliations:** 1 Department of Hematology, CHOC Chidren's Hospital, Orange, CA, 92868, USA; 2 Department of Infectious Disease, CHOC Children's Hospital, Orange, CA, 92868, USA; 3 Department of Radiology, CHOC Children's Hospital, Orange, CA, 92868, USA; 4 Department of Pathology, CHOC Children's Hospital, Orange, CA, 92868, USA

**Keywords:** innate immunity, toll like receptors, interleukin-1 receptor-associated kinase-4, case report

## Abstract

*Pseudomonas aeruginosa* ( *P. aeruginosa*) is an aerobic Gram-negative bacterium that is implicated in the development of severe systemic infections among pediatric patients.  It is identified in hospitalized chronically ill pediatric patients in association with genitourinary, respiratory tract, and skin or soft tissue infections as well as severe and life-threating infection including sepsis.  A variety of immunologic mechanisms play a vital role in the host defense mechanisms against invasive infections with *P. aeruginosa*. Rarely, specific inborn errors of immune function are implicated in deficiencies that predispose to invasive infections with *P. aeruginosa*.  Innate immune function including germ-line encoded pattern recognition receptors such as toll-like receptors (TLRs) and their downstream signaling is vital in the host defense against *P. aeruginosa* through the generation of antimicrobial peptides, cytokines/chemokines, and shaping of adaptive immune responses. Herein, we describe a previously healthy two-year-old female with an invasive skin, soft tissue, and central nervous system infection secondary to *P. aeruginosa*.  The invasive nature of this infection prompted a careful evaluation for an inborn error of immunity. Decreased cytokine response to agonists of TLRs was documented. Targeted sequencing of interleukin-1 receptor-associated kinase (IRAK)-4 documented a homozygous deletion of exons 8-13 consistent with IRAK-4 deficiency.  This report provides a vital educative message in the existing scientific literature by underscoring the importance of considering inborn errors of immunity in all patients with severe *P. aeruginosa* infections.  Functional assessments of immune function often in combination with sequencing can accurately assign a diagnosis in a timely fashion allowing for definitive treatment and the use of necessary supportive care.

## Introduction

*Pseudomonas aeruginosa* ( *P. aeruginosa*) is an opportunistic aerobic Gram-negative bacterial pathogen associated with a variety of genitourinary (UTI), pulmonary as well as skin and soft tissue infections (SSTI) in hospitalized pediatric patients often in association with significant morbidity ^1^. Rarely, *P. aeruginosa* can be associated with severe and life-threatening infections among children without previously recognized associated risk factors ^2^. In these cases, it is vital to consider the possibility of an underlying inborn error of immunity. Invasive *P. aeruginosa* infections have been described in the setting of inborn errors of immunity including antibody deficiencies (agammaglobulinemia) Bruton (Bruton agammaglobulinemia), combined immunodeficiency disorders (severe combined immunodeficiency, ataxia telangiectasia), defects of phagocytes (chronic granulomatous disease, leukocyte adhesion deficiency), defects in actin-polymerization (Wiskott-Aldrich syndrome, MKL1-deficiency), chronic neutropenia and innate immunity including defects in canonical NFKB-signaling (e.g., NEMO/NFKBIA) as well as those that impair the downstream signaling of toll-like receptors (TLRs), such as defects in interleukin-1 receptor-associated kinase (IRAK)-4 and myeloid differentiation factor 88 (MyD88) ^2, 3^. We describe the presence of an invasive soft tissue and central nervous system infection with *P. aeruginosa* in a previously healthy two-year-old female which prompted evaluation for an inborn error of immunity.

## Case report

**Figure 1.**
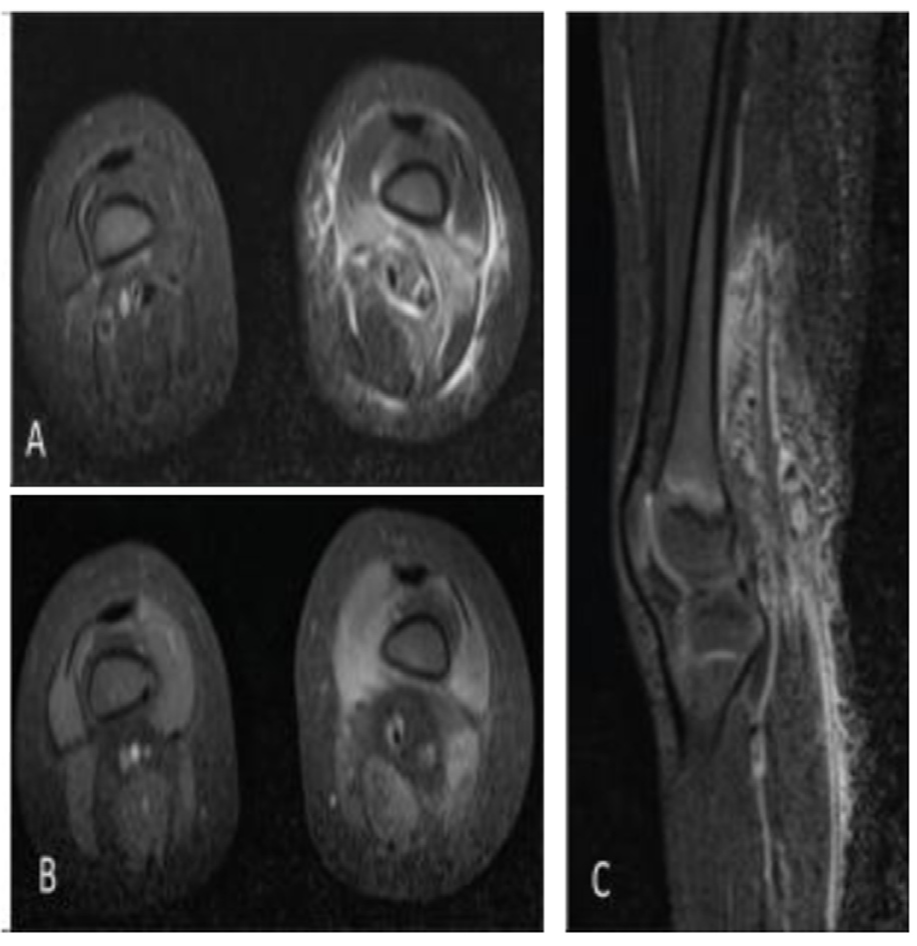
Axial ( **A**) and sagittal ( **C**) Short-TI Inversion Recovery (STIR) magnetic resonance imaging (MRI) images of the bilateral lower extremities demonstrate edematous signal involving the left lower extremity vastus medialis, vastus intermedius, soft tissues of the popliteal fossa, and overlying subcutaneous soft tissues. Axial fat-saturated T1-weighted postcontrast MRI of the bilateral lower extremities ( **B**) demonstrates associated enhancement of the vastus medialis, vastus intermedius, and soft tissues of the popliteal fossa. Findings are compatible with myositis with overlying cellulitis.

A previously healthy two-year-old Hispanic female was evaluated for left hip and knee pain associated with a fever and a refusal to ambulate. She had been given cephalexin for presumed insect bites on her back and legs. Her past medical history was non-contributory. She was fully immunized. Her family history was negative for consanguinity and inborn errors of immunity. She was afebrile and her physical examination demonstrated a 3 cm circular erythematous lesion on her mid left back which was non-indurated. Her left knee was minimally swollen, warm to touch, and non-erythematous. Her hips and knees remained in flexion with demonstrated resistance to knee extension. A nodular 1.5 cm mass in her popliteal fossa was also documented with tenderness upon palpation and she was unable to walk. Her laboratory investigation demonstrated a white blood cell count of 17.2 × 10 ^3^/uL (normal range: 3.9-13.7), Hgb 10.4 g/dL (normal range:10.2-15.4), platelets 319 × 10 ^3^ (normal range: 150-450), 64% neutrophils (normal range: 25-72), 26% lymphocytes (normal range: 24-71), 9% monocytes (normal range: 0-14), 0.6% eosinophils (normal range: 2-10), 0.3% basophils (normal range: 0-2), erythrocyte sedimentation rate (ESR) 126 mm/hr (normal range: 0-20), and C-reactive protein (CRP) 68.8 mg/L (normal range: 0-10). A blood culture was negative. Magnetic resonance (MR) imaging of her lower extremity demonstrated myositis and fasciitis involving the soft tissues of the distal left thigh, but no abscess ( Figure 1A–C). Likely pathogens included methicillin sensitive *Staphylococcus aureus*, methicillin resistant *Staphylococcus aureus*, and group A *Streptococcus.*

**Figure 2.**
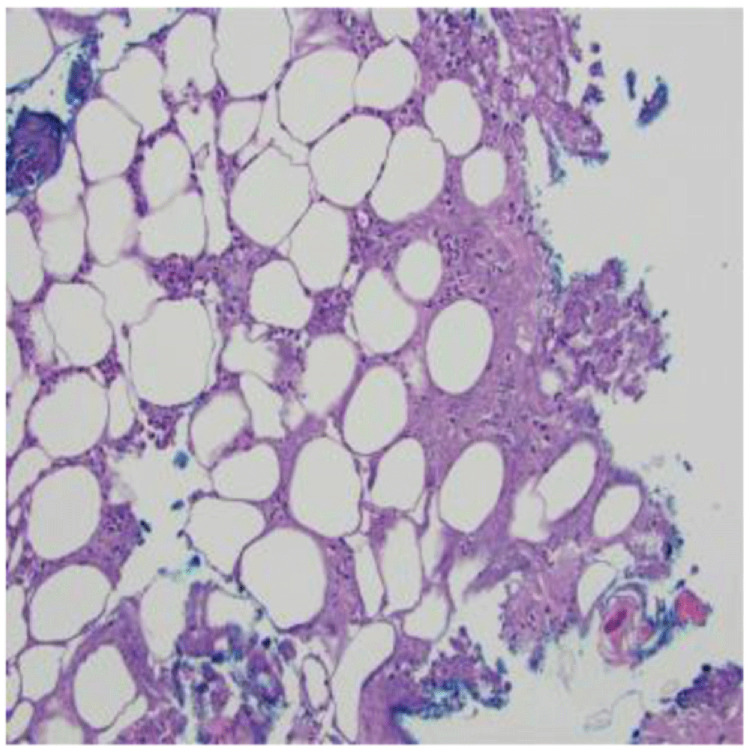
Skin punch biopsy demonstrates acute suppurative panniculitis with suppurative necrosis (20×, Hematoxylin and Eosin).

Empiric antibiotic therapy began with vancomycin (60 mg/kg/day intravenously divided every 6 hours for 3 days) and ceftriaxone (75 mg/kg/day intravenously every 24 hours for 3 days) which was then transitioned to cefazolin (100 mg/kg/day intravenously divided every 8 hours for 7 days), clindamycin (30 mg/kg/day intravenously divided every 8 hours for 7 days), amoxicillin-clavulanate (50 mg/kg/day orally divided every 12 hours for 4 days), and then to linezolid (30 mg/kg/day intravenously divided every 8 hours for 6 days) due to a lack of clinical and laboratory improvement. A lesion on her back was biopsied and demonstrated acute suppurative panniculitis and suppurative necrosis ( Figure 2). Cultures were obtained with growth of *P. aeruginosa* and a 3-week course of cefepime (150 mg/kg/day intravenously divided every 8 hours) for SSTI was completed. Clinical and laboratory improvement occurred as demonstrated by an ESR 57 mm/hr (normal range: 0–20) and CRP <5 mg/L (normal range: 0–10). The patient was discharged while awaiting results of a workup for an inborn error of immunity.

**Figure 3.**
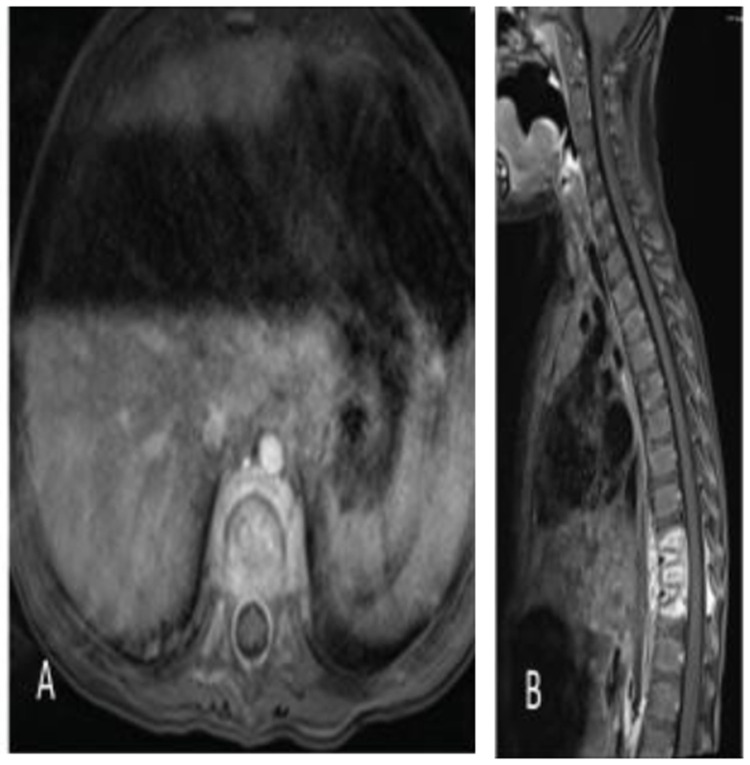
Axial ( **A**) and sagittal ( **B**) fat-saturated volumetric interpolated breath-hold examination (VIBE) postcontrast magnetic resonance imagery (MRI) images of the thoracic spine demonstrate multilevel thoracic osteomyelitis and discitis with associated epidural and paravertebral abscesses.

She then returned approximately 3 weeks later with malaise, an inability to stand upright, irritability, and pain on palpation of her back as well as a refusal to ambulate. On re-admission she was afebrile, and her laboratory investigation demonstrated an ESR 89 mm/hr (normal range: 0-20), and CRP <5 mg/L (normal range: 0-10). MR imaging of her spine demonstrated enhancement of T9-T11 with an epidural abscess ( Figure 3A–B). A culture was obtained by computed tomography (CT)-guided needle aspiration and empiric therapy with meropenem (120 mg/kg/day intravenously divided every 8 hours for 2 days) was begun. Growth of *P. aeruginosa* was documented and a 6-week course of cefepime (150 mg/kg/day intravenously divided every 8 hours) was completed. Clinical improvement occurred; however, her laboratory investigation demonstrated an ESR 80 mm/hr (normal range: 0–20) and CRP <5 mg/L (normal range: 0–10) at the completion of therapy.

**Figure 4.**
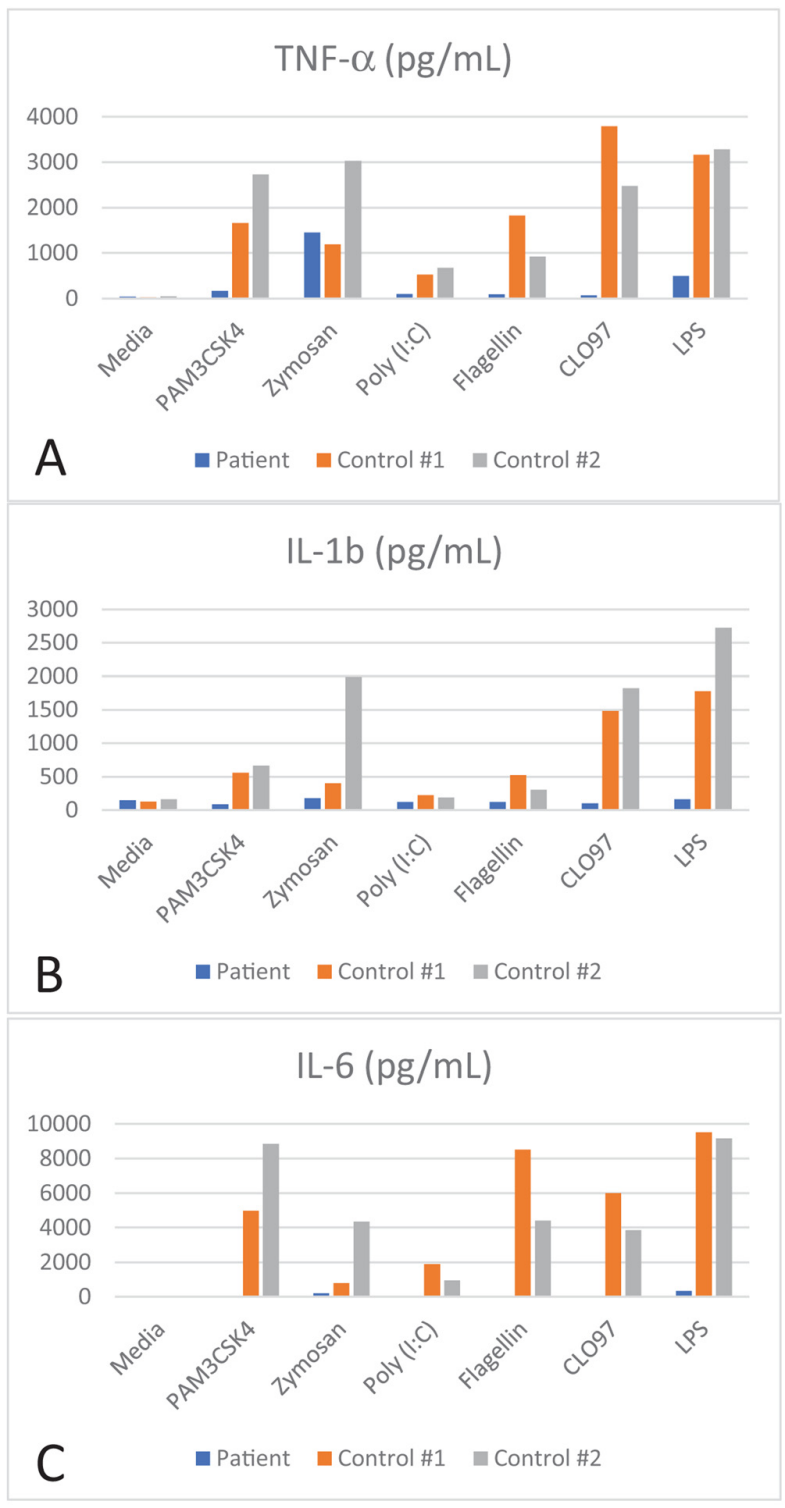
Decreased cytokine response to toll-like receptor (TLR) agonists: **A**) TNF alpha, **B**) IL-1b, and **C**) IL-6 was measured following peripheral blood mononuclear cell (PBMC) stimulation with PAM3CSK4 (TLR2 and TLR1), zymosan (TLR2 and TLR6), Poly (I:C) (TLR3), flagellin (TLR 5), CLO97 (TLR7 and TLR8), and LPS (TLR4).

Secondary to the invasive nature of her *P. aeruginosa* infection, evaluation for an inborn error of immunity was completed. A normal neutrophil oxidative burst was noted. Additional laboratory assessments included a serum IgG 1331 mg/dL (normal range: 407-1009), IgA 85 mg/dL (normal range: 22-220), an elevated IgM 362 mg/dL (normal range: 43-163), and a IgE 3.8 kU/L (normal range: <97). Antibody responses documented a non-protective *Haemophilus influenza* type b antibody titer (0.30 mcg/L), a tetanus antibody titer which was protective (0.21 IU/mL), and pneumococcal titers which were protective (> 1.3 mcg/L) for nearly all serotypes covered by Prevnar 13. Lymphocyte immunophenotyping demonstrated a CD3+ count of 1340/ uL (normal range: 1484-5327), CD4+ count of 633 / uL (normal range: 733-3181), CD8+ count of 628/ uL (normal range: 370-2555), CD19+ count of 493/ uL (normal range: 370-2306), CD16/56+ count of 109/uL (normal range: 43-526). Decreased cytokine response to TLR agonists were documented by a commercial lab (ARUP Laboratories) using peripheral blood mononuclear cells ( Figure 4). LPS-induced CD62L shedding in neutrophils is an additional potential screening test for TLR defects, but was not completed in this case ^4^. Targeted sequencing of IRAK-4 and MyD88 was performed. A homozygous deletion of exons 8-13 was documented in IRAK-4 consistent with a diagnosis of IRAK-4 deficiency. There were no siblings; however, it is important to underscore the importance of screening other family members for the same pathogenic variants.

Following the diagnosis of IRAK-4 deficiency she was start on prophylaxis with intravenous immunoglobulin (0.5 g/kg/dose intravenously every 4 weeks) as well as amoxicillin (250 mg orally each day). From her diagnosis at 2 years of age until 4 years of age she continued to experience infrequent infectious complications including a urinary tract infection ( *Escherichia coli*), left knee swelling in association with a abscess (methicillin-susceptible *Staphylococcus aureus*), and a single admission for fever, cough, and post-tussive emesis. She is now 6 years of age and doing well without any recent infectious complications. She remains compliant with her prophylaxis therapy with intravenous immunoglobulin therapy and amoxicillin which she has tolerated without complications.

## Discussion

Detection of lipopolysaccharide and flagellin by TLRs results in the elaboration of pro-inflammatory cytokines ^3^. TLRs possess an intracellular domain known as the Toll–IL-1R domain (TIR). Upon activation of TLRs, the recruitment of TIR-containing cytosolic adaptors such as MyD88 occurs. The adaptor MyD88 then recruits cytosolic kinases, including the IRAK complex. The IRAK complex includes two kinases including IRAK-4 and two non-catalytic subunits. This results in the activation of downstream effectors including nuclear factor κB (NF-κB) and mitogen-activated protein kinases which support the synthesis of pro-inflammatory cytokines and chemokines, such as IL-1β, -6, -8, and -12 and tumor necrosis factor alpha.

IRAK-4 deficiency is an autosomal recessive disorder which requires that affected patients have homozygous or compound heterozygous mutations in the IRAK-4 ^4– 6^. IRAK-4 deficient patients typically have normal basic immunological evaluations ^4– 6^. Importantly, inflammatory responses are markedly blunted as demonstrated by the severe and life-threatening invasive bacterial infection with *P. aeruginosa* in our patient accompanied by an absence of CRP elevation. In these patients, CRP concentrations can be strikingly misleading as IRAK-4 deficient patients demonstrate impairment in the ability to increase CRP concentrations and to mount fever responses.

Among IRAK-4 deficient patients with invasive bacterial infection, *S. pneumoniae* is the most frequently (~50% of episodes) implicated organism ^6^. *S. aureus* and *P. aeruginosa* are less frequently (~20% of episodes) implicated organisms ^6^. Patients with IRAK-4 deficiency may also experience a variety of minor non-invasive bacterial infections such as upper respiratory tract infections (otitis, sinusitis, pharyngitis) as well as SSTI (furunculosis, folliculitis cellulitis) ^6^. Once again, the most frequently implicated organisms in these non-invasive bacterial infections among IRAK-4 deficient patients are *S. pneumoniae, S. aureus*, and *P. aeruginosa.*

Careful institution of aggressive supportive care measures is necessary for IRAK-4 deficient patients. Vaccines should include conjugated and nonconjugated vaccines for *S. pneumoniae* and *N. meningitidis. Haemophilus influenzae* type b conjugated vaccine should also be provided. Lifelong antibiotic prophylaxis with cotrimoxazole in combination with penicillin should be administered. Prophylaxis with intravenous or subcutaneous IgG should also be provided as a significant proportion of IRAK-4 deficient patients have impaired responses to glycans. Empiric parenteral antibiotic therapy against *S. pneumoniae*, *S. aureus*, and *P. aeruginosa* is critical whenever an infection is suspected or if the patient develops a fever. Inflammatory markers such as CRP should be considered unreliable. Importantly, patients may die from invasive bacterial infection despite prophylaxis even in the absence of fever or laboratory evidence of inflammation. The long-term prognosis of IRAK-4 deficient patients is positive as the risk of invasive infections tend to improve with age.

Although the occurrence of invasive *P. aeruginosa* infections in IRAK-4 deficiency is not novel, there is a vital and important educative message that bears repeating. Astute clinical judgment is necessary in the evaluation of patients with a potential inborn error of immunity. Clinical findings such as severe infections with *P. aeruginosa* should support the consideration of inborn errors of immunity including IRAK-4 deficiency.

## Data availability

All data underlying the results are available as part of the article and no additional source data are required.

## Consent

Written informed consent for publication of their clinical details and/or clinical images was obtained from the parent of the patient.
