# Supplementary material for: Case Report: Pseudomonas can take a toll on a patient
Source: F1000Res. 2021 Sep 21;10:526. Originally published 2021 Jul 1. [Version 2] doi: 10.12688/f1000research.53424.2 (PMC8323073; doi:10.12688/f1000research.53424.2)
Supplement: Peer review history

# Peer review: Reviewer response for version 1

**DOI:** 10.5256/f1000research.56805.r88939

**Published:** 2021-07-29

**Authors:** Shanmuganathan Chandrakasan

**Affiliations:** 1 Division of Bone Marrow Transplant, Aflac Cancer and Blood Disorders Center, Children's Healthcare of Atlanta, Emory University, Atlanta, GA, USA

Very well-written case report of a child with IRAK4 deficiency. I have no major comments, few minor comments include:

- Briefly discuss the role of CD62L shedding assay as another screening test. ^1^
- Explain briefly why there is a need for Ig replacement in a disorder that is mainly myeloid lineage being affected- it might be of interest to the readers
- A line on long term prognosis might be helpful- the risk of invasive infection tend to improve with age

Are enough details provided of any physical examination and diagnostic tests, treatment given and outcomes?

Yes

Is the case presented with sufficient detail to be useful for other practitioners?

Yes

Is sufficient discussion included of the importance of the findings and their relevance to future understanding of disease processes, diagnosis or treatment?

Yes

Is the background of the case’s history and progression described in sufficient detail?

Yes

Reviewer Expertise:

Immunobiology of Immune dysregulation disorders; HSCT and gene therapy for immune defects

I confirm that I have read this submission and believe that I have an appropriate level of expertise to confirm that it is of an acceptable scientific standard.

## References

[1] : A fast procedure for the detection of defects in Toll-like receptor signaling.*Pediatrics*.2006;118(6) : 10.1542/peds.2006-18452498-50310.1542/peds.2006-184517142536

# Response

**Published:** 2021-08-18

**Authors:** David K. Buchbinder

**Affiliations:** CHOC Children's Hospital, USA

Reviewer #2:

 Comment #1:

 Briefly discuss the role of CD62L shedding assay as another screening test.

 Response #1:

 Thank you for this excellent and important suggestion. Additional discussion regarding the role of the CD62L shedding assay as a potential screening test has been added. The suggested reference has also been added.

 von Bernuth H, Ku CL, Rodriguez-Gallego C, Zhang S, et al.: A fast procedure for the detection of defects in Toll-like receptor signaling.Pediatrics. 2006; 118 (6): 2498-503.

 Comment #2:    

 Explain briefly why there is a need for Ig replacement in a disorder that is mainly myeloid lineage being affected- it might be of interest to the readers

 Response #2:

 Thank you for this suggestion. Additional discussion focusing on explaining why immunoglobulin prophylaxis has a role in IRAK4 deficiency has been added. 

 Comment #3:

 A line on long term prognosis might be helpful- the risk of invasive infection tend to improve with age

 Response #3:

 We apologize for this omission and agree that it is important to comment on the long-term prognosis of patients with IRAK4 deficiency.

# Peer review: Reviewer response for version 1

**DOI:** 10.5256/f1000research.56805.r88936

**Published:** 2021-07-05

**Authors:** David Hagin

**Affiliations:** 1 Sackler Faculty of Medicine, Tel Aviv University, Tel Aviv, Israel

I had a pleasure reading this case report. The authors nicely describe a rare case of IRAK-4 deficiency in a female patient with invasive pseudomonal infection.

 The case report highlights several important issues. First, and most important, unusual and/or severe presentation should trigger further evaluation for possible underlying inborn errors of immunity (IEI). In this case, invasive infection and failure to respond to antibiotics raised this concern.

 Next, the case report highlights additional important laboratory findings, such as normal CRP levels in the presence of severe infection. In that regard, this case report shows that ‘normal’ CRP should not always be considered a reassuring sign, but rather the opposite, and in some cases could suggest a failure to mount a required inflammatory response.

 Finally, the case report describes a very elegant stepwise work-up approach, starting with identifying ‘clinical clues’ (such as normal CRP), followed by ordering the correct diagnostic assay, and finally performing targeted sequencing of a **single** candidate gene (sadly, not that common anymore).

 There are several minor points I would like to suggest:

1. Decreased cytokine response to TLR agonists: Was that performed by a commercial lab? If so, I think I would mention it so readers would know that it can be ordered. Also, which cells were used (fibroblasts/PBMCs)?
2. Were the authors able to test for LPS-induced CD62L shedding in neutrophils?
3. I don’t know if the patient has siblings, but perhaps the Discussion should include a recommendation to screen other family members for the same pathogenic variants.

Are enough details provided of any physical examination and diagnostic tests, treatment given and outcomes?

Yes

Is the case presented with sufficient detail to be useful for other practitioners?

Yes

Is sufficient discussion included of the importance of the findings and their relevance to future understanding of disease processes, diagnosis or treatment?

Yes

Is the background of the case’s history and progression described in sufficient detail?

Yes

Reviewer Expertise:

Inborn Errors of Immunity/Primary Immunodeficiency Disorders

I confirm that I have read this submission and believe that I have an appropriate level of expertise to confirm that it is of an acceptable scientific standard.

# Response

**Published:** 2021-08-18

**Authors:** David K. Buchbinder

**Affiliations:** CHOC Children's Hospital, USA

Reviewer #1:

 Comment #1:

 Decreased cytokine response to TLR agonists: Was that performed by a commercial lab? If so, I think I would mention it so readers would know that it can be ordered. Also, which cells were used (fibroblasts/PBMCs)?

 Response #1:

 Thank you for this comment. The cytokine response to TLR agonists was performed by a commercial lab. We agree that this is important to point out for readers. Moreover, peripheral blood mononuclear cells were utilized for testing. This was also pointed out to help other readers/clinicians that may be facing similar clinical situations. 

 Comment #2:

 Were the authors able to test for LPS-induced CD62L shedding in neutrophils?

 Response #2:

 We did not test for LPS-induced CD62L shedding in neutrophils. As suggested by Reviewer #2, additional discussion regarding the role of the CD62L shedding assay as a potential screening test has been added.

 Comment #3:

 I don’t know if the patient has siblings, but perhaps the Discussion should include a recommendation to screen other family members for the same pathogenic variants.

 Response #3:

 The patient does not have any siblings, but we agree that this is an important point to make from a clinical perspective. Additional discussion regarding the importance of screening other family members for the same pathogenic variants has been added.
